# Pulsed Field Ablation for Persistent Superior Vena Cava: New Solution for an Old Problem

**DOI:** 10.1016/j.jaccas.2022.01.015

**Published:** 2022-03-02

**Authors:** Shota Tohoku, Boris Schmidt, Stefano Bordignon, Shaojie Chen, Fabrizio Bologna, Lukas Urbanek, Francesco Pansera, K.R. Julian Chun

**Affiliations:** a Cardioangiologisches Centrum Bethanien (CCB), Frankfurt Academy For Arrhythmias (FAFA); Abteilung für Kardiologie, Medizinische Klinik III, Agaplesion Markus Krankenhaus, Frankfurt am Main, Germany; b Universitätsklinikum Frankfurt, Medizinische Klinik 3-Klinik für Kardiologie, Frankfurt, Germany; c Die Sektion Medizin, Universität zu Lübeck, Lübeck, Germany

**Keywords:** ablation, atrial fibrillation, electrophysiology, imaging, AF, atrial fibrillation, PFA, pulsed field ablation, PLSVC, persistent left superior vena cava, PN, phrenic nerve, PV, pulmonary vein

## Abstract

Persistent left superior vena cava (PLSVC) is a known arrhythmogenesis site in patients with atrial fibrillation. However, the optimal PLSVC isolation approach has remained unclear because of the potential risk of complications. The current study reports 2 cases of successful electrical PLSVC isolation using pulsed field ablation. (**Level of Difficulty: Intermediate.**)

## Central Illustration

**Figure:**
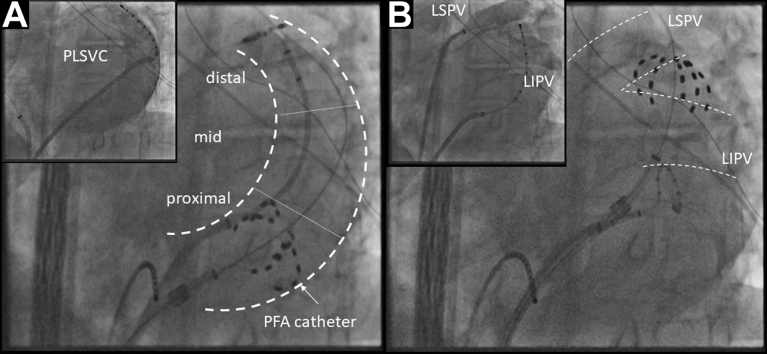

Persistent left superior vena cava (PLSVC) is one of the most common thoracal venous anomalies in adult cardiac malformations, irrespective of congenital malfunction.^1^ Higher incidence of arrhythmogenesis from PLSVC has been reported in patients with atrial fibrillation (AF), indicating that electrical PLSVC isolation is important in AF ablation.^2^^,^^3^ However, the optimal approach for PLSVC isolation has remained unclear owing to the potential risk of complications.^4^^,^^5^

**Learning Objectives**

• To report the first clinical experience of successful electrical isolation of PLSVC with pulsed field ablation in patients with atrial fibrillation.
• To suggest a potential role of pulsed field ablation as an approach for PLSVC ablation reducing the complication risk.

Pulsed field ablation (PFA) is a new method of myocardial-specific ablation in which direct current electric energy—electric field—is applied to cells and disrupts cell membranes by creating pores.^6^ In a preclinical study, the optimized electric field was reported to affect the myocardium selectively.^7^

FARAPULSE (Boston Scientific, European CE-Mark [not yet approved in the United States]) consists of the 12-F catheter (FARAWAVE, Boston Scientific) with 5 splines, carrying 4 electrodes, each navigated over the wire via a steerable sheath.^8^ The catheter shape can be changed from starfish-like to flower-like, according to targeted anatomy.

The combined concepts of PFA and the catheter form may be an applicable approach for PLSV ablation. Herein, 2 cases of successful electrical isolation of PLSVC with PFA are reported.

## Case 1

**Figure 1:**
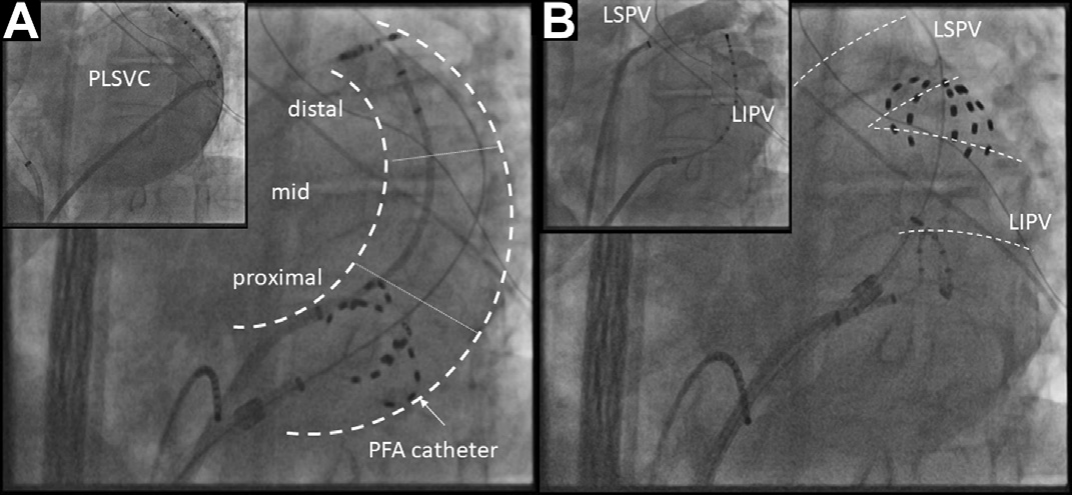
Fluoroscopic Image of Catheter Positioning during PLSVC Isolation in Case 1 **(A)** Selective angiography of PLSVC with left anterior oblique (LAO) 40° in Case 1 and the fluoroscopic image of PFA catheters positioned in the proximal PLSVC. **(B)** The successful elimination of PLSVC potential was achieved at the distal PLSVC on the level between left superior and inferior PVs. PFA = pulsed field ablation; PLSVC = persistent left superior vena cava; PV = pulmonary vein.

**Figure 2:**
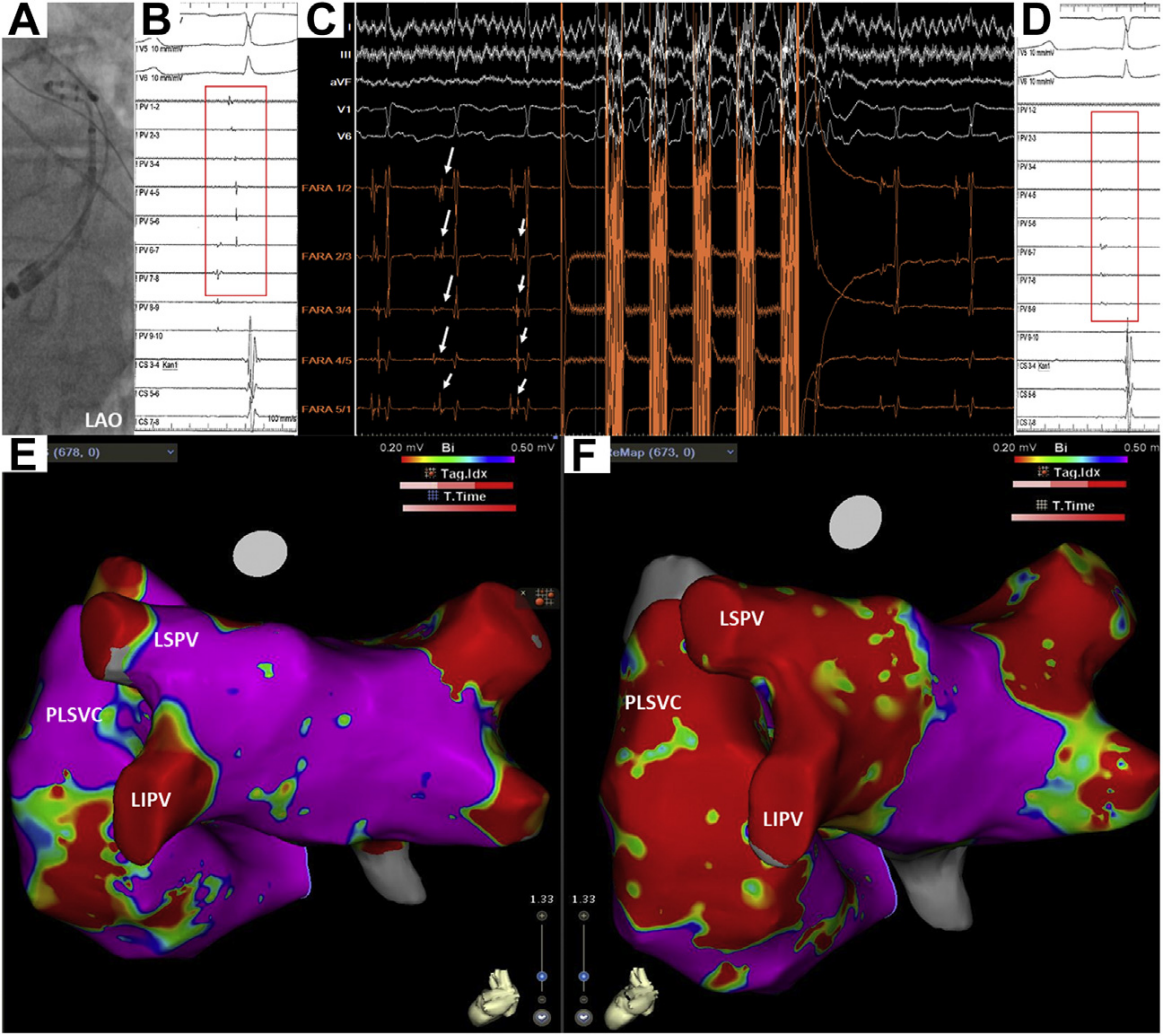
Pre- and Post-PLSVC Isolation in Case 1 **(A)** Fluoroscopic view of a spiral diagnostic catheter positioned in the distal PLSVC with LAO 40°. **(B)** Intracardiac electrogram showing PLSVC potential before application. **(C)** Intracardiac electrogram showing the elimination of local PLSVC signals by an application in Case 1. **(D)** Intracardiac electrogram showing eliminated PLSVC potential after application. **(E, F)** Comparison of the left atrial voltage maps before **(left)** and after **(right)** ablation with the CARTO 3D mapping system with posterior-anterior oblique. Abbreviations as in Figure 1.

A 73-year-old male patient with a history of hypertension presented with worsening dyspnea and lower-extremity edema. The electrocardiogram indicated AF. A transesophageal echocardiogram excluded structural heart disease and showed a dilated ostium of the coronary sinus; a patent foramen ovale of 5 mm; and atresia of the right superior vena cava, which indicated a PLSVC. The patient was admitted to undergo an AF ablation after appropriate clinical management of heart failure. The procedure was planned with 3-dimensional (3D) mapping-system guidance (CARTO, Biosense Webster). The strategy was switched to PFA, using a 35-mm FARAWAVE with a single transseptal approach because of the difficult transseptal puncture after PLSVC angiography (Figure 1A). The successful isolation of all 4 pulmonary veins (PVs) was achieved with an output of 1.9 Kv. Thereafter, frequent extra systole beats with the earliest activation site at the PLSVC were observed. Four applications, with an output of 1.9 Kv, were delivered through the catheter in the starfish-like form, from the proximal to distal PLSVC, after confirming the left phrenic nerve (PN) capture, by pacing from the 35-mm catheter at 12 m A/2.0 ms in PLSVC. PN capture was observed during applications at mid-distal PLSVC. The last application on the level between the left superior and inferior PVs successfully eliminated the electric PLSVC potential (Figures 1A, 1B, 2A to 2C). A 3D voltage map was delineated at the end of the procedure (Figures 2D and 2E).

## Case 2

**Figure 3:**
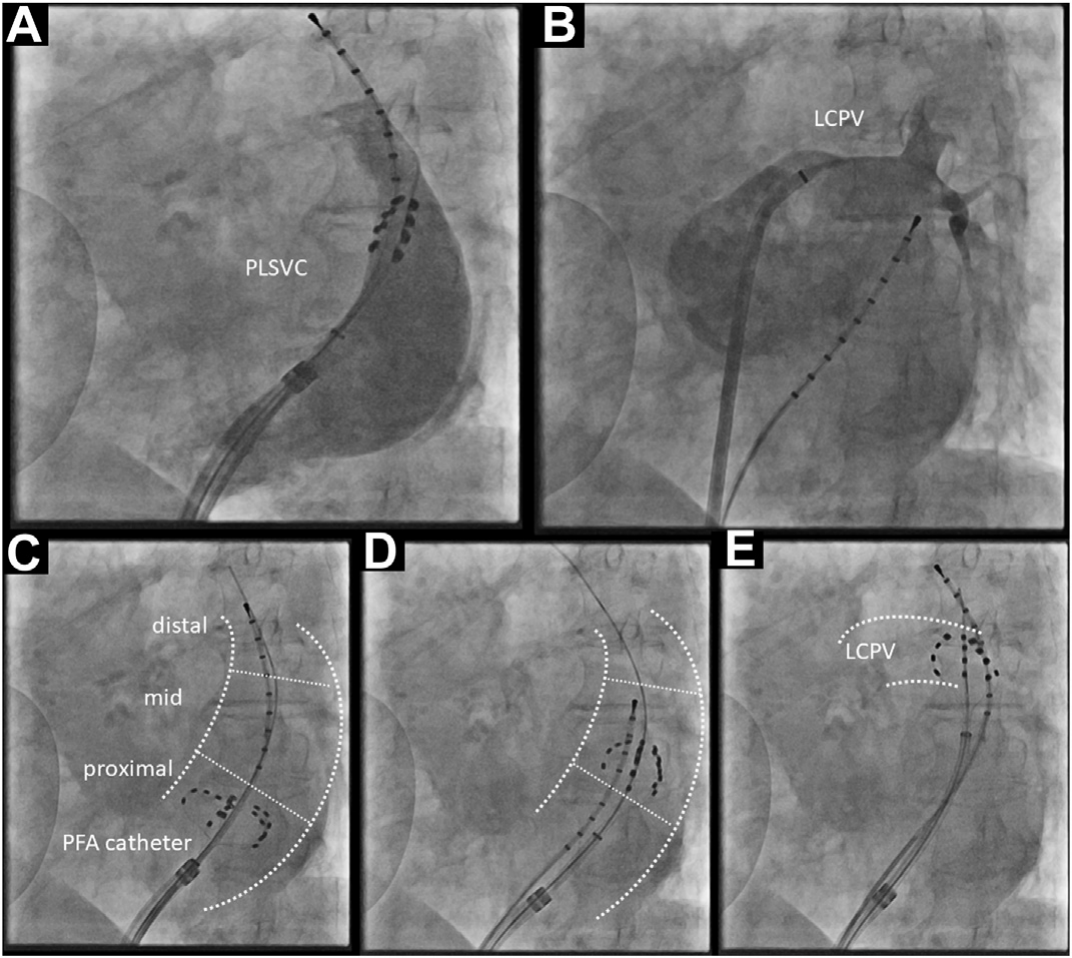
Fluoroscopic Image of Catheter Positioning During PLSVC Isolation in Case 2 **(A,B)** Selective angiographies of PLSVC and left common PV with LAO 40° in Case 2. **(C to E)** The fluoroscopic images of PFA catheters positioned in the proximal **(C)**, middle **(D)**, and distal **(E)** PLSVC. The successful elimination of PLSVC potential was obtained with an application at the distal PLSVC on the level of the left common PV. Abbreviations as in Figure 1.

**Figure 4:**
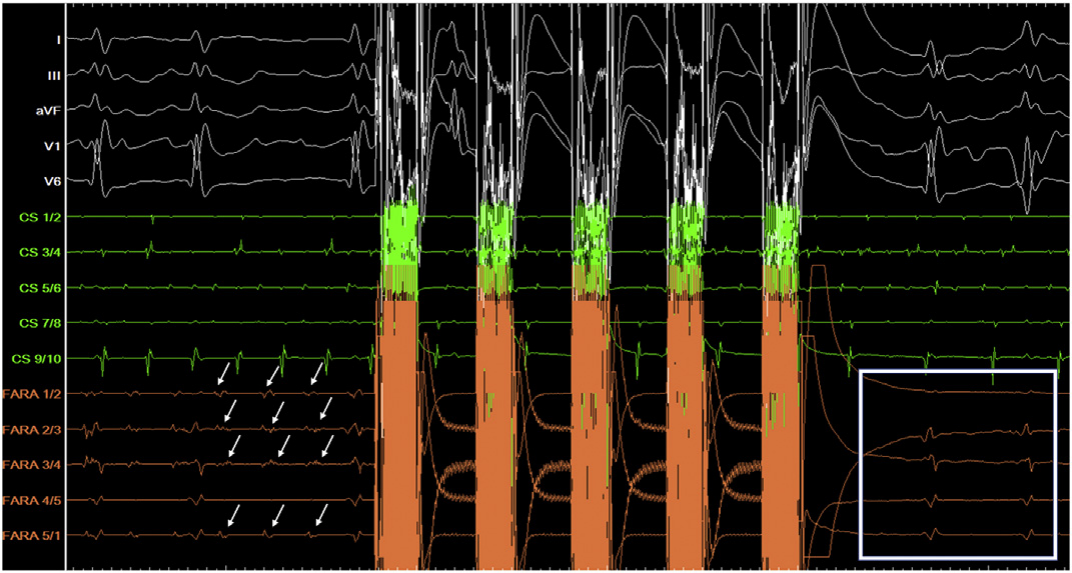
Intracardiac Electrogram Showing the Elimination of Local PLSVC Signals by Application in Case 2

A 78-year-old female patient with previously diagnosed sick sinus syndrome type 3 subsequent to paroxysmal AF was admitted for worsening palpitation. Catheter ablation was indicated after excluding other structural heart diseases. A transesophageal echocardiogram showed a dilated ostium of the coronary sinus and a common ostium of lateral PVs. The procedure was started with a selective angiography of PVs and PLSVC (Figures 3A and 3B) under fluoroscopic guidance with a 35-mm catheter. Frequent extra systole beats with the earliest activation site at the PLSVC were observed, even after the successful isolation of all 4 PVs. Three applications with an output of 1.9 Kv were delivered in the starfish-like pose at the proximal, middle, and distal PLSVC after confirming the left PN capture by pacing from the 35-mm catheter at 12 mA/2.0 ms in PLSVC (Figures 3C to 3E). PN capture was observed during applications at the mid-distal PLSVC. The last application at the level of the left common PV successfully eliminated the electric PLSVC potential (Figure 4).

Left atrium (LA)-PLSVC disconnection was confirmed with the entrance block based on real-time isolation of PLSVC potential in both cases. However, neither diaphragmatic dysfunction nor ST-segment change on 12-lead electrogram was observed.

## Discussion

This study is believed to be the first description of the successful PLSVC isolation with PFA. The major thoracic veins, with their specific electrical properties, have an established role in AF genesis and maintenance.^1^, ^2^, ^3^ The left superior cardinal vein is part of such primitive common cardinal vein and remains as PLSVC in case of failure to regress during embryologic development.^1^, ^2^, ^3^ Thus, the clinical effect of eliminating the electrical potential of PLSVC has been previously reported.^2^^,^^3^ However, the safety and feasibility of the procedure have been under debate owing to the potential risk of complications (eg, cardiac tamponade, PN palsy, coronary arterial injury, and superior vena cava syndrome caused by vascular stenosis). Some reports referred to non-negligible rates of complications irrespective of energy source.^4^^,^^5^

The biophysical concept of PFA offers nonthermal lesion formation through the mechanism of irreversible electroporation and has recently been applied to catheter ablation.^6^^,^^7^ In PFA, the lesion boundary is determined by the irreversible electroporation threshold value of the electric field in the tissue of interest. The preclinically examined optimal threshold value, depending on voltage and waveform details, was much lower for myocardial cell compared with those for neuroglial cell, vascular smooth cell, and red blood cell in animal models.^6^^,^^7^ Therefore, the basic biophysical theory may potentially be applied to PLSVC ablation.

LA-PLSVC disconnection was observed only at the distal portion of the PLSVC in both cases. This may be explained by the enlarged proximal part of PLSVC. The area of proximal PLSVC adhering to the myocardial sleeve may be too large to be isolated with lateral part of the catheter in the starfish-like pose. Moreover, electrical connection between PLSVC and LA was present only along one-half of its circumference after the middle part.^2^ Therefore, PLSVC isolation using the current 35-mm catheter seems attainable at the mid-distal portion.

PLSCV ablation succeeded without any procedural relevant complications in the 2 current cases. No routine coronary angiogram was performed in the absence of ST change. However, a correlation between PFA and coronary vasospasm was suggested in a single case report by Gunawardene et al.^9^ The procedural feasibility including lesion durability should be investigated with further studies.

## Conclusions

Two cases of successful PLSVC isolation were reported in patients with AF using the pulsed field ablation device without observing signs of procedure relevant complications, which suggested the possibility of PFA as an alternative approach to PLSVC ablation, reducing the risk of complications.

## Funding Support and Author Disclosures

All patients in this report provided written informed consent before undergoing the ablation procedure. This report was approved by the Institutional Review Board. The authors have reported that they have no relationships relevant to the contents of this paper to disclose.
